# Parturition and the Principles and Practice of Obstetrics

**Published:** 1850-01

**Authors:** 


### Part 2.—Reviews and Notices of New Works.

### ARTICLE I.

*Parturition and the Principles and Practice of Obstetrics.*— By W. TYLER SMITH, M.D., London. Lecturer on Obstetrics in the Hunterian School of Medicine. pp. 375. Duodecimo. Philadelphia. Lea & Blanchard. 1849.— (From the Publishers, and for sale by S. C. Griggs & Co., Chicago.)

This is a work devoted principally to the application of the doctrines of Reflex Physiological Action as taught by Marshall Hall, to Obstetrics. In this application, the author claims originality, which, if not true as regards the idea, is certainly so in reference to the extent to which these speculations have been carried.

The work is arranged in lectures, of which there are twenty-six with an appendix.

The style of the author, although occasionally obscure, is generally good, and free from the bombast that has blemished the writings of some on the same subject, and which is particularly objectionable in works of a scientific and practical character.

Our author introduces his work by drawing a comparison between British and Continental Obstetrical Practice; the the result of which, according to his showing, is, that Protestant is decidedly preferable to Roman Catholic influence in controlling practice. This, he thinks, is proved by the difference in the choice between the life of the mother, and that of the child, when one or the other must be sacrificed.

Dr. Smith is decidedly British in his views, and thinks it very doubtful whether the introduction of the opinions and improvements of other countries into British Obstetrics, has not been of decided disadvantage. But we think his boast of a preference for craniotomy in England, if we refer to the statistics of these and forceps cases from that country and from France, by no means establishes the superiority of British humanity and civilization. And the want of success in England, in the Cæsarean operation, may as fairly be considered a cause of its dread as religious opinion—especially among those who have no religious feeling whatever. But again, it may be said that English want of success depends much on the dread of its performance, as delay will always diminish the chances of succes.

Dr. Tyler Smith is decided in the expression of his opinions on most of the subjects of which he treats; and however we may differ with him on some points of importance in practice, and notwithstanding he may not have convinced us of the truth of his reflex doctrines in all respects, still we are highly pleased with many parts of the work. His arguments against the separation of the placenta in unavoidable hemorrhage, as recommended by Prof. Simpson, are forcible, and coincide with our own convictions of propriety. The chances for preserving the life of the mother would appear equally as good in the old practice of turning to deliver, while those of the child are beyond comparison better. In fact, the practice of separating the placenta offers scarcely any chance to the life of the child, and of course it would require positive proof, of not only its decided advantage to the mother, but of its necessity to her safety to justify the practice.

The author is especially severe on Midwives, or female practitioners of the Obstetric Art, who, according to his account, are fast going out of fashion in England. He thinks the "*sage femmes*" of France superior to the English Midwives.

but thinks the manner of their advertisement throws ridicule on this department of medicine in that country.

The following passage will give a specimen of the author's style, and shows "*la mode de Paris*" for announcing the pretensions of the female practitioner.

"Even in the most aristocratic quarters of Paris—the Rue and Faubourg St. Honorè, for instance—the '*sonnette de sage-femme*,' or the door-bell of the midwife, may be seen with a miniature picture of the smart midwife herself, in oil colors, over it, in the act of presenting a fine baby to all comers. Or there may be observed paintings of more pretension, giving the passer-by the penetralia o the lying-in room, and containing full length portraits of the accoucheuse and the everlasting baby, with an interesting view of the lady-accouchèe in bed in the back-ground; the not over-delicate painting by Rubens, in the gallery of the Louvre, of the delivery of Marie de Medici, reduced to common-place—the *sage-femme* doing the work of the angels of that celebrated artist."

We had intended to give a somewhat extended analysis of the work in the present number of the Journal, but must defer this for want of time to give it the attention it demands.

